# A light-gated cation channel with high reactivity to weak light

**DOI:** 10.1038/s41598-023-34687-7

**Published:** 2023-05-10

**Authors:** Shoko Hososhima, Shinji Ueno, Satoshi Okado, Ken-ichi Inoue, Masae Konno, Yumeka Yamauchi, Keiichi Inoue, Hiroko Terasaki, Hideki Kandori, Satoshi P. Tsunoda

**Affiliations:** 1 Department of Life Science and Applied Chemistry, Nagoya Institute of Technology, Showa-ku, Nagoya, Aichi 466-8555 Japan; 2 OptoBioTechnology Research Center, Nagoya Institute of Technology, Showa-ku, Nagoya, Aichi 466-8555 Japan; 3 Department of Ophthalmology, Nagoya University Graduate School of Medicine, 65 Tsurumai-cho, Showa-ku, Nagoya, Aichi 466-8550 Japan; 4 Department of Ophthalmology, Hirosaki University Graduate School of Medicine, 5 Zaifu-cho, Hirosaki, Aomori 036-8562 Japan; 5 Primate Research Institute, Kyoto University, Inuyama, Aichi 484-8506 Japan; 6 Present Address: The Institute for Solid State Physics, The University of Tokyo, 5-1-5 Kashiwanoha, Kashiwa, Chiba 277-8581 Japan; 7 Present Address: PRESTO, Japan Science and Technology Agency, 4-1-8 Honcho, Kawaguchi, Saitama 332-0012 Japan

**Keywords:** Biophysics, Neuroscience

## Abstract

The cryptophyte algae, *Guillardia theta*, possesses 46 genes that are homologous to microbial rhodopsins. Five of them are functionally light-gated cation channelrhodopsins (GtCCR1-5) that are phylogenetically distinct from chlorophyte channelrhodopsins (ChRs) such as ChR2 from *Chlamydomonas reinhardtii.* In this study, we report the ion channel properties of these five CCRs and compared them with ChR2 and other ChRs widely used in optogenetics. We revealed that light sensitivity varied among GtCCR1-5, in which GtCCR1-3 exhibited an apparent EC_50_ of 0.21–1.16 mW/mm^2^, similar to that of ChR2, whereas GtCCR4 and GtCCR5 possess two EC50s, one of which is significantly small (0.025 and 0.032 mW/mm^2^). GtCCR4 is able to trigger action potentials in high temporal resolution, similar to ChR2, but requires lower light power, when expressed in cortical neurons. Moreover, a high light-sensitive response was observed when GtCCR4 was introduced into blind retina ganglion cells of *rd1*, a mouse model of retinitis pigmentosa. Thus, GtCCR4 provides optogenetic neuronal activation with high light sensitivity and temporal precision.

## Introduction

Channelrhodopsins (ChRs) are directly light-gated ion channels found in chlorophyte and cryptophyte alga as well as in the relative alga^1–4^. Cation-conducting ChRs (CCRs) such as ChR2 from *Chlamydomonas reinhardtii*, conduct cations such as H^+^, Na^+^, K^+^, and Ca^2+^. High-resolution X-ray structures of CCRs revealed details of their molecular architecture and provided insight into the photoactivation and ion conduction pathway^5,6^. Anion-conducting ChRs (ACRs) were subsequently discovered and their molecular architecture has been solved^7,8^.

**Figure 1:**
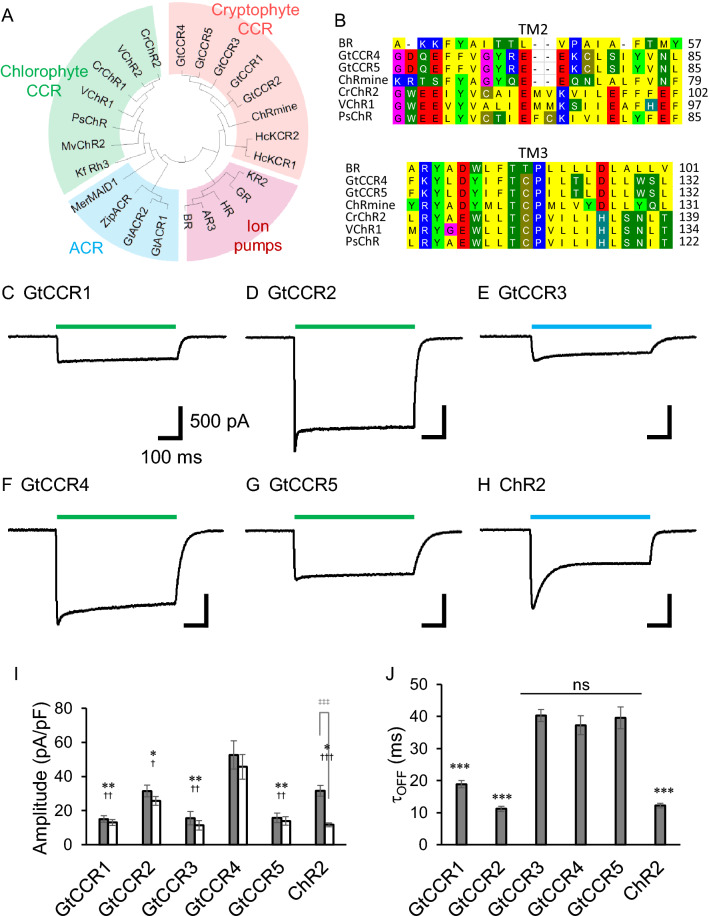
Comparison of photocurrent properties of GtCCR1-5. (**A**) Phylogenetic tree of ion-transporting rhodopsins. (**B**) Amino acid alignment of TM2 and TM3 region of CCRs. *BR* bacteriorhodopsin, *GtCCR4* GtCCR5 and ChRmine belong to cryptophyte CCR, whereas CrChR2, VChR1 and PsChR are involved in chlorophyte CCR. (**C**–**H**) Representative photocurrent traces of GtCCR1–5 and ChR2. Membrane voltage was clamped at − 60 mV. 530 nm light (**C**,**D**,**F**,**G**) and 470 nm light (**E**,**H**) at 2.7 mW/mm^2^ were illuminated. (**I**) Photocurrent amplitude at − 60 mV by strong light at 2.7 mW/mm^2^. GtCCR1, 2, 4, and 5 were illuminated by 530 nm light, and GtCCR3 and ChR2 by 470 nm light. Gray bar: peak component; white bar: steady state component. (**J**) Comparison of the channel-closing kinetics after shutting-off light (τ-off).

Genetic delivery of opsin-encoding genes to cells and tissues originally light insensitive tuned into light-sensitive. Thus, both CCRs and ACRs have been applied in the manipulation of action potentials in light-insensitive cells and tissues with unprecedented spatio-temporal precision, initiating a new research field, optogenetics^9–12^. In 2016 and 2017, Govorunova et al. and we independently identified phylogenetically distinct cation ChRs from the cryptophyte algae *Guillardia theta*^13,14^. These cation ChRs (i.e. GtCCRs) are more homologous to haloarchaeal rhodopsins, such as proton-pumping bacteriorhodopsin (BR), than to chlorophyte CCRs, including ChR2 from *Chlamydomonas reinhardtii* (ChR2). GtCCRs have conserved characteristic amino-acid residues for unidirectional proton transfer, including D85, T89 and D96 in TM3 of BR (DTD motif), while ChR2 possesses ETH or ETK at the corresponding positions (Fig. 1B)^1^. On the other hand, a characteristic glutamic acid in TM2 (E90 in ChR2), which is crucial for channel gating and ion selectivity, is not conserved in GtCCRs^15^. ChR2 possesses a so-called DC pair which consists of C128 and D156 bridged by hydrogen bonds through a water molecule^6^. Mutations in these positions dramatically extend the lifetime of the channel^11,16,17^. The DC pair is not found in the GtCCRs. Thus, overall, sequence patterns separate these cryptophyte CCRs from chlorophyte channels. Recently, two groups independently reported the molecular architecture of cryptophyte CCR (ChRmine), revealing a trimeric assembly with large vestibules in the monomer, presumably forming a channel pore^18,19^. As we reported previously, GtCCR4 shows high conductance for monovalent metal cations, i.e. negligible permeability for proton and divalent cations, including Ca^2+^^20^. Here, we compared the ion channel properties of five GtCCRs and chlorophyte channels more comprehensively. Considering its high light sensitivity, we tested the feasibility of GCCR4 for optical manipulations of cultured neurons.

Vision restoration by optogenetic therapy has been attempted for years. Retinitis pigmentosa (RP) is mainly caused by the loss of photoreceptor cells (rods and cones) in the outer layer of retina, and thus results in blindness^21^. However, the retina still retains the light-insensitive bipolar cells and ganglion cells in the inner layer. Introducing ChR2 or its variants into bipolar cells or ganglion cells (RGCs) by an adeno-associated viral vector enables these cells to become light-sensitive, thereby restoring visual function^22–25^. The first therapy was reported in 2021, in which the patient partially recovered their visual function after expressing ChrimsonR, a red-shifted variant of ChR^26^. The patient was able to perceive, locate, count and touch different objects. The report suggests that optogenetic therapy is a promising way to cure RP patients, although the patient needed goggles to stimulate the retina. This could be due to the limited light sensitivity of ChrimsonR. To test the applicability of GtCCR4, we here demonstrate the restoration of light sensitivity of the retina in an RP mouse model.

## Results

### Basic characterization

Figure 1A shows a phylogenetic tree of ion-transporting rhodopsins, including cation channels (CCR), anion channels (ACR) and ion pumps. The CCRs form two clusters, chlorophyte CCRs and cryptophyte CCRs. Interestingly, chlorophyte CCRs branch from ACRs, whereas cryptophyte CCRs are more homologous with the pump-type rhodopsins. The amino acid alignment of TM2 and TM3 in Fig. 1B shows marked differences between the chlorophyte and cryptophyte CCRs. In particular, the position of the proton donor of BR Asp 96 in TM3 is conserved in cryptophyte CCRs, GtCCR4, GtCCR5 and ChRmine, whereas this position is replaced by His or Lys in chlorophyte CCRs.

Electrophysiological studies of GtCCR1-4 have already been reported^13,14,20,27^. Here, we measured the cation channel activities of all GtCCRs including newly identified GtCCR5 in parallel. ChR2 was also measured in comparison. We subcloned all CCR genes into the eYFP 3.0 vector and expressed them in ND7/23 cells by a conventional transfection method. The expression of these channels was visualized by the eYFP-tag under a fluorescent microscope. We then carried out photocurrent measurement by a patch-clamp. A green LED (530 nm) at 2.7 mW/mm^2^ was used for GtCCR1, 2, 4 and 5, while the same power of a blue LED (470 nm) was used to illuminate GtCCR3 and ChR2 because their maxima of their action spectrum are blue-shifted. Figure 1C–H show representative photocurrents of GtCCR1-5 and ChR2 at − 60 mV. We observed a light-induced current from cells expressing GtCCR1, GtCCR2 and GtCCR3, reproducing the findings of a previous study by Govorunova et al.^13^. The photocurrents of GtCCR4 exhibited a peak current (I_p_) upon illumination which decayed into a steady state level (I_ss_), i.e. 20% of inactivation. The photocurrent of GtCCR5 with 530 nm light is similar to that of GtCCR4 but shows a smaller inactivation (10%). In contrast, the photocurrent of ChR2 shows large inactivation during illumination (60%). The current–voltage relationship (I–V plot) of GtCCR1-5 and ChR2 are summarized in Fig. S1. All the CCRs showed inward-rectification. Given that the wavelength dependency of GtCCR5 was unknown, we measured its action spectrum. The spectrum is almost identical to that of GtCCR4, peaking at around 525 nm (Fig. S2).

The photocurrent density (pA/pF) of all CCRs at a light intensity of 2.7 mW/mm^2^ was compared (Fig. 1I). The photocurrent of GtCCR1, 2, 3 and 5 were relatively low, ranging from about 15 to 30 pA/pF at − 60 mV. The photocurrent amplitude from GtCCR4 exceeded 53 ± 8.3 pA/pF (I_p_) decaying into I_ss_ of 46 ± 7.2 pA/pF, while ChR2 also showed an I_p_ of 32 ± 3.2 pA/pF but with a large inactivation decaying into about 12 ± 1.1 pA/pF (I_ss_). Channel-closing kinetics after light-off was compared (Fig. 1J). The τ_off_ of GtCCR1 and GtCCR2 was 19 ± 1.1 ms and 11 ± 0.8 ms, which is similar to that of ChR2 (12.2 ± 0.69 ms), while GtCCR3, 4 and 5 showed a slower off-kinetics of about 40 ms (40 ± 1.9, 37 ± 3.0 and 40 ± 3.4 ms respectively).

### Light-power dependency

**Figure 2:**
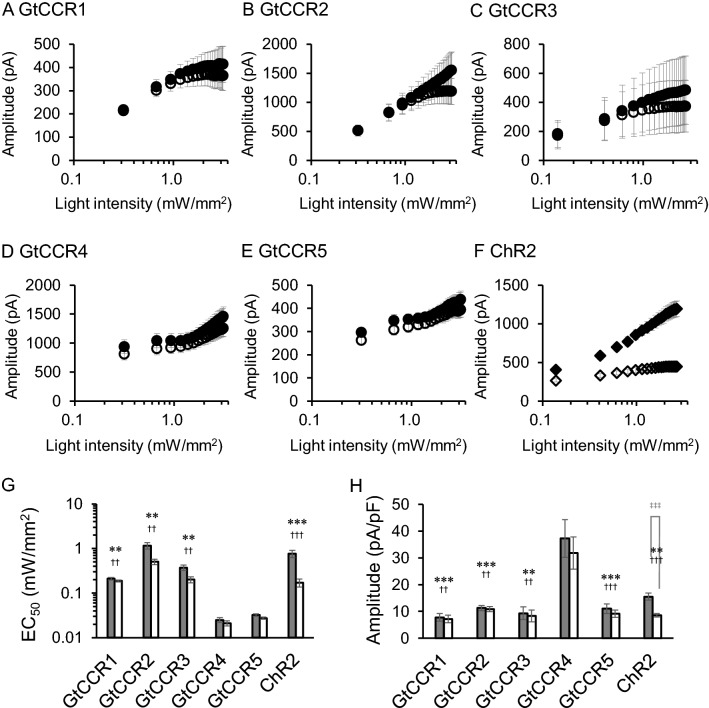
Light power dependency of photocurrent amplitude by GtCCRs and ChR2. Photocurrent amplitude at − 60 mV was plotted as a function of light power. 530 nm light (**A**,**B**,**D**,**E**) and 470 nm light (**C**,**H**) were illuminated. Filled circle: peak component; open circle: steady state component. (**G**) The half saturation maximum (EC_50_) of the peak current (gray bar) and the steady state current (white bar) are shown. (**H**) Photocurrent amplitude at − 60 mV induced by weak light illumination (0.3 mW/mm^2^).

We previously reported the high light sensitivity of GtCCR4 compared to ChR2^20^. Here, we compared all five GtCCRs and ChR2. Both I_p_ and I_ss_ were recorded in the range up to 3 mW/mm^2^ (Fig. 2A–F). The photocurrent amplitude from GtCCR1-3 and ChR2 grows as a typical sigmoidal curve for both the initial peak and the steady state components (Fig. 2A–C,F). However, the results of GtCCR4 and 5 show unusual power dependency (Fig. 2D,E). The photocurrent first became saturated at about 0.5–1.0 mW/mm^2^, followed by the second growth from 1 to 3 mW/mm^2^. We further tested the recordings of GtCCR4 and GtCCR5 with even weaker light power (Fig. S3). In the range up to 0.25 mW/mm^2^, both GtCCR4 and GtCCR5 showed a typical sigmoidal curve and already became saturated with about 0.1 mW/mm^2^, whereas ChR2 did not become saturated in this range.

We determined the EC_50_ of light activation by a hyperbolic fitting (Fig. 2G). Unexpectedly, the EC50 showed a large variation among the five GtCCRs. GtCCR1 and 2 had a large EC_50_ of 0.21 ± 0.013 mW/mm^2^ and 1.2 ± 0.19 mW/mm^2^ respectively, while 0.37 ± 0.050 mW/mm^2^ was obtained for GtCCR3. However, GtCCR4 and 5 showed two EC_50_ values, ones with a very small EC50 of 0.025 ± 0.0032 mW/mm^2^ and 0.032 ± 0.002 mW/mm^2^, respectively, whereas the second EC50 values were 2.0 ± 0.56 mW/mm^2^ and 1.6 ± 0.29 mW/mm^2^. These results suggest that both GtCCR4 and GtCCR5 are highly light-sensitive CCRs compared to the other CCRs, when the first EC50 is compared. But the second EC50 of GtCCR4 and GtCCR5 is taken into account, the EC_50_ of the all GtCCRs are in the same range. Figure 2H shows photocurrent amplitude at − 60 induced by weak light (0.3 mW/mm^2^), indicating that the largest current was observed in GtCCR4-expressing cells.

**Figure 3:**
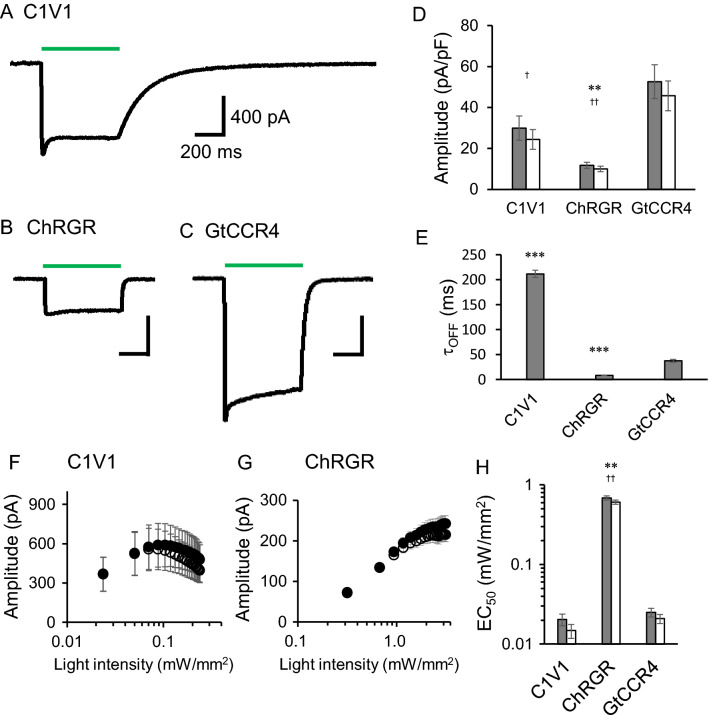
Comparison of photocurrent properties of GtCCR4 and green light-activating ChRs. Representative photocurrent traces of C1V1 (**A**), ChRGR (**B**) and GtCCR4 (**C**). Membrane voltage was clamped at − 60 mV. 530 nm light at 2.7 mW/mm^2^ was illuminated. (**D**) Comparison of the photocurrent amplitude from three ChRs. Photocurrent amplitude provided by strong light at 2.7 mW/mm^2^. Gray bar: peak component; white bar: steady state component. (**E**) Comparison of the channel-closing kinetics after shutting-off light (τ-off). (**F**,**G**) Light power dependency of photocurrent of C1V1 and ChRGR. Filled circle: peak component; open circle: steady state component. (**H**) Light sensitivity (EC_50_) of the three ChRs. Gray bar: peak component; white bar: steady state component.

We further investigated light dependency. There are ChRs that absorb green-yellow light with good performance in optogenetics research, such as C1V1 and ChRGR^28–30^. Thus, these CCRs were expressed in ND7/23 cells and green light-induced currents were compared under the same experimental condition. Figure 3A–C show representative photocurrent traces of C1V1, ChRGR and GtCCR4 at − 60 mV with the same light intensity (2.7 mW/mm^2^). Current shape and amplitude varied among them (Fig. 3D). Photocurrent decay after light-off was markedly slow in C1V1 (Fig. 3A). The τ_off_ values are depicted in Fig. 3E. τ_off_ reached 200 ms in C1V1. ChRGR exhibited a τ_off_ of 20 ms, the fastest among the three CCRs, while the value of GtCCR4 was 30 ms. On the other hand, GtCCR4 exhibited the highest current amplitude (Fig. 3D). ChRGR exhibited the smallest photocurrent under the same condition.

Next, we compared light sensitivity of the three CCRs in a weak light range. Figure S4 shows representative photocurrent traces in various light intensities. The photocurrent of C1V1 grew slowly upon illumination and also showed a slow decay after shutting off light. The photocurrent of C1V1 and ChRGR in various light intensities was plotted (Fig. 3F,G). For GtCCR4, refer to Fig. 2D and Fig. S3A. The photocurrent amplitude from C1V1 already became saturated at about 0.1 mW/mm^2^ (Fig. 3F), which is similar to GtCCR4 (Fig. S3A). However, the current amplitude decreased as light intensity increased between 0.1 and 0.24 mW/mm^2^. ChRGR reached a plateau at about 2 mW/mm^2^ (Fig. 3G). Of note, the range of light intensity of two panels in Fig. 3F is one order of magnitude different. The half-saturation maximum of the photocurrent (EC_50_) was estimated (Fig. 3H). C1V1 exhibited an EC_50_ of 0.020 ± 0.0034 mW/mm^2^, while the EC_50_ of ChRGR was 0.69 ± 0.044 mW/mm^2^. This result indicates that the light sensitivity of C1V1 and GtCCR4 (the first EC50) are one order of magnitude higher than that of ChR2 and ChRGR.

### Optogenetics application of GtCCR4 for neuronal excitation

**Figure 4:**
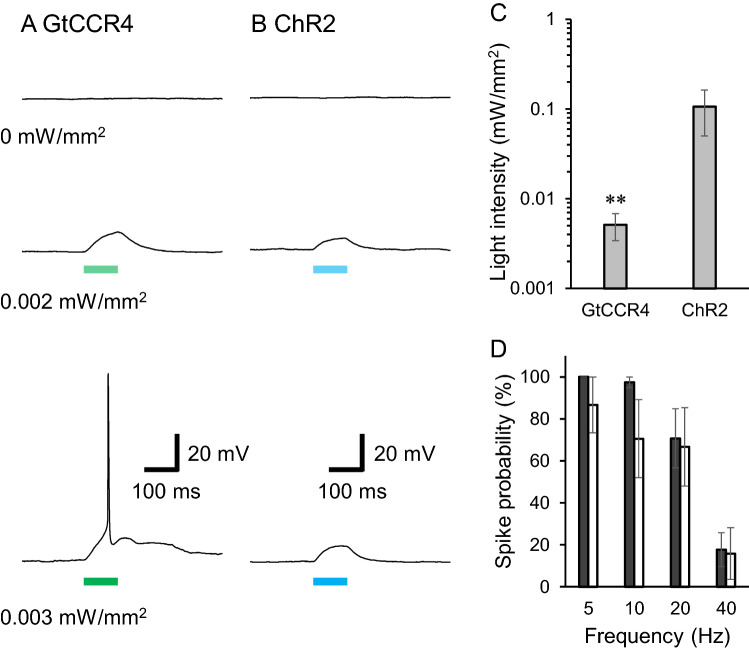
Optical stimulation of cultured neurons by weak light. Representative action potentials evoked by various light power are shown in (**A**): GtCCR4-expressing neuron, (**B**): ChR2–expressing neuron. 530 nm light (**A**), and 470 nm light (**B**) were applied on neurons for 100 ms as indicated by colored bars. (**C**) Minimum light intensities for triggering an action potential in each neuron. (**D**) Spike probabilities at 5, 10, 20 and 40 Hz illumination are shown. Black bar: GtCCR4-; white bar: ChR2-expressing neurons. 530 nm and 470 nm light were applied to GtCCR4 and ChR2 neurons, respectively.

We then examined the potential of GtCCR4 as an optogenetic tool for reliably eliciting the action potential of pre-cultured neurons. In particular, this was assessed under weak light. Rat-cortical neurons were isolated and transfected with a vector carrying a neuron-***specific protomer CaMKII. As a reference, we expressed ChR2. Transfected cells were identified by the fluorescent proteins under a fluorescent microscope, and the membrane potential was monitored by a patch-clamp with a current-clamp configuration. Figure 4A,B show representative membrane potentials from neurons expressing GtCCR4 (Fig. 4A), and ChR2 (Fig. 4B) under weak light. Even though all neurons were slightly depolarized at 0.002 mW/mm^2^ light, no action potential was observed. However, the membrane could be depolarized to a subthreshold level, and an action potential was successfully elicited at only 0.003 mW/mm^2^ in GtCCR4-expressing neurons (Fig. 4A), whereas no action potentials were triggered in ChR2-expressing neurons upon illumination with 470 nm light (0.001–0.003 mW/mm^2^) (Fig. 4B). Action potentials were triggered at more than 0.04 mW/mm^2^ in ChR2-expressing neurons (Fig. S5). As light power was increased, multiple spikes were triggered in GtCCR4- and ChR2-expressing neurons (Fig. S6). We further explored the ability of GtCCR4 to activate neurons at various light intensities. Figure 4C shows minimum light intensities for triggering action potentials in GtCCR4- and ChR2-expressing neurons. On average, 0.0051 ± 0.0017 mW/mm^2^ light (530 nm) was required for GtCCR4 neurons, while more than 0.11 ± 0.056 mW/mm^2^ light (470 nm) was needed for ChR2 neurons. This indicates that GtCCR4 is a potential optogenetics tool that works efficiently under weak light. These results from neurons are consistent with the experiments from ND7/23 cells shown in Fig. 2.

One of the advantages of the use of ChRs for neuronal spiking is the high-time resolution which allows precise temporal manipulation of action potentials. Here, we probed the frequency response of light-spike coupling with GtCCR4-expressing neurons (Fig. 4D, Fig. S7). The light intensity was fixed at 2.7 mW/mm^2^. A single spike was reliably elicited at 5 or 10 Hz for GtCCR4-expressing neurons (100 and 97% respectively) for 3 s, while accuracy was lower in ChR2-expressing neurons (87 and 71% respectively). When illuminated at 20 Hz, spike probability decreased to 67–71% for the GtCCR4- and ChR2-expressing neurons, and even less at 40 Hz (< 20%). This result indicates that GtCCR4 can mediate spiking more precisely at 5 and 10 Hz compared to ChR2, similar to ChR2 at 20–40 Hz.

### Restoration of light response in *rd1* mouse retina

**Figure 5:**
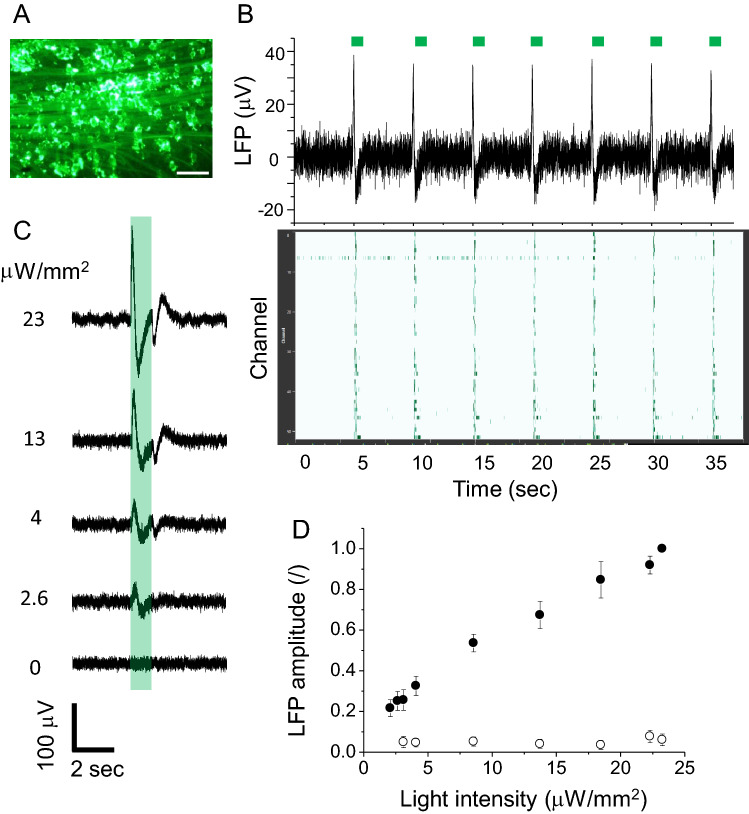
Restoration of light response in the blind retina from *rd1* mouse expressing GtCCR4. (**A**) Fluorescent image of *rd1* retina expressing GtCCR4-veuns. scale bar: 100 μm. (**B**) Upper: representative LFP trace of multi electrode array recording from retina expressing GtCCR4. Retina was photo-stimulated for 1 s every 5 s as indicated by green bars. Lower: raster plot of unit responses of multi electrode array (64 channels). (**C**) Comparison of LFP amplitude under various light intensities from GtCCR4-expressing retina. Retina was stimulated by 530 nm light for 1 s. Light powers are indicated on each trace. (**D**) Light power dependency of on-response of the LFP from GtCCR4-expressing retinas (filled circle) and PBS-injected retinas (open circle). Retinal was stimulated by 530 nm light.

Finally, we tested whether light response was restored in *rd1* mouse retina after GtCCR4 expression. After 4 weeks of virus infection, the retina was isolated to assess its response to light by using a multi electrode array system (Fig. 5). Successful infection and expression in retina RGC were confirmed by the venus fluorescence (Fig. 5A). Figure 5B (upper) shows a typical local field potential (LFP) trace of a single channel of the multi array upon brief light stimulations indicated by green bars (for 1 s × 7 times). Figure 5B (lower) shows a raster plot of unit responses. The light-induced LFP was rapidly evoked and reliably repeated every 5 s without significant desensitization of the amplitude. This implies that the response did not weaken with repeated optical stimulation. Figure 5C shows representative LFP traces upon illumination of various light intensities. The GtCCR4-expressing retina was highly light sensitive. Indeed, light pulses as low as 2.6 μW/mm^2^ triggered ~ 20% of the maximum response tested in this experiment (23 μW/mm^2^). We analyzed the light power dependency of the on-responses of the LFP (Fig. 5D). The on-response exhibited an almost linear relation to the light intensities tested (Fig. 5D, filled circle), whereas the PBS-injected retina showed no response (Fig. 5D, open circle). These results suggest that GtCCR4-expressing blind retina (RGC) restored high light sensitivity..

## Discussion

In this study, we comprehensively compared the photocurrent properties of cryptophyte CCRs, specifically photosensitivity and kinetics. All GtCCRs (GtCCR1-5) exhibited substantial channel activity without large inactivation which is observed in the ChR2 photocurrent. On the other hand, light sensitivity (EC_50_) was markedly different among them, with GtCCR4 and 5 having a highly sensitive component (EC50 = 0.026–0.032 mW/mm^2^) and a less sensitive EC_50_ (1.6–2.0 mW/mm^2^), whereas the EC_50_ values of GtCCR1-3 were similar to that of ChR2. It is important for photosensitive cells or organs to respond to both weak and strong light conditions, for which human eyes contain rod and cone visual cells, respectively, while higher plants contain phototropin1 and phototropin2, respectively^31,32^. Among the five known GtCCRs, only GtCCR3 absorbs blue light (460 nm) while the remaining GtCCRs absorb green light (500–540 nm). Although physiological roles of GtCCRs are unknown, it might be that GtCCR4/5 or GtCCR1/2 respond to weak and strong light, respectively. These characteristics of GtCCRs are possibly advantageous for the survival of *G. theta*, which lives in brackish water where environment conditions such as nutrition, salt, and light may vary dramatically.

Possessing CCRs with different light sensitivities might help to widen the photo-response range in the native organism, similar to that of animal eyes and higher plants. However, when the molecular mechanism is taken into account, there are prominent differences among them. Light-intensity dependent responses in animal eyes and higher plants originate from signal amplification processes based on enzymatic reactions. In the case of human eyes, rod and cone visual pigments activate a G-protein transducin (Gt), which further activates phosphodiesterase (PDE), leading to a closed c-GMP gated channel in visual cells^33^. Two-step amplification processes by Gt and PDE largely distinguish the sensitivities of rod and cone cells, and thus a wide dynamic range (~ 10^6^ photons μm^–2^ ) is possible for human eyes^34^. A similar enzyme-based amplification system exists in higher plants^35^. On the contrary, *Chlamydomonas reinhardtii* has a signal transduction system, where ChR2 and ChR1 mediate phototaxis to weak and strong light respectively, although the photosensitivities of ChR2 and ChR1 do not differ significantly^36^. On the other hand, the present results suggest that only GtCCR proteins are responsible for different photo sensitivities, as assessed by EC_50_. The EC_50_ of GtCCR2 (1.2 mW/mm^2^) and that of GtCCR4 (the first EC_50_ = 0.025 mW/mm^2^) differ 48-fold despite the high amino acid sequence homology. Thus, understanding the molecular mechanism of these molecules is of great interest.

The photosensitivity of a light-gated channel can be characterized by the efficiency of channel opening and channel open time, the latter being measured as the tau (τ)-off value. High light-sensitive ChRs were reported in chlorophyte ChRs, such as step function opsins, whose photocycles are prolonged with a corresponding increase in τ-off of 1000–10,000 times^16,17^. Low EC_50_ values of C1V1 are also due to large τ-off values (Fig. 3E). In contrast, in the case of GtCCR1-5, the τ-off values are in the same range (11–40 ms), suggesting that the channel open time is not an essential factor determining the difference in photosensitivity. Although a longer τ-off of GtCCR4 (40 ms) than of GtCCR2 (11 ms) surely contributes to the difference of EC_50_ to some extent, the 48-fold difference might not be fully explained. The efficiency of channel opening can be characterized by absorptivity (molar coefficient), quantum yield of retinal isomerization, and conversion ratio of the primary intermediate into a channel-opening intermediate. Similar characteristics are known for homologous microbial rhodopsins, among which quantum yields of retinal isomerization vary to a large extent^37,38^. Nevertheless, quantum yield is typically in the range of 0.2–0.6, still making it hard to explain the 48-fold difference in EC_50_ between GtCCR2 and GtCCR4. An attractive hypothesis is to postulate a cooperative effect, where a photoactivated GtCCR4 opens another protein in an oligomer, allowing apparent quantum yield to become > 1. Although this idea is entirely speculative, a recent cryo-EM structure of ChRmine, a homologous CCR to GtCCR4, suggested the presence of an auxiliary ion-pathway within the trimer interface^18^. The molecular mechanism of channel activation should be studied by using purified proteins, which will be our future focus.

A comparison of the channel properties of GtCCR4 with chlorophyte CCRs (C1V1 and ChRGR), which are widely used in optogenetics, shows that GtCCR4 is also high light-sensitive even with a relatively rapid τ-off (Fig. 3). This inspired us to test GtCCR4 for its application in optogenetics. We revealed high light sensitivity of GtCCR4 for photostimulation of cultured neurons, even though the temporal resolution for neuronal activation was as high as that of ChR2 (Fig. 4). Thus, use of GtCCR4 would be advantageous in optogenetics when light power is limited (e.g. a target in deep tissue) and a moderately high temporal stimulation is required. Nevertheless, it should be noted that a much faster CCRs are needed for certain application e.g. in fast-spiking interneurons or in auditory neurons. Introducing mutations into GtCCR4 for faster kinetics is one of our next studies.

Attempts have been made in ophthalmology to restore visual function by using ChRs for many years. One of the challenges in optogenetics gene therapy is to enhance light sensitivity since the reported cases pointed out low light sensitivity when ChRs were introduced into the RGC or the bipolar cells of deficient retina^22,23,25,39^. Restoring light sensitivity in a blind mouse retina is a promising use of GtCCR4 in the restoration of visual function in higher mammals. Further improvements in light sensitivity would be needed for gene therapy to restore visual function in room light or in a dim light environment.

## Materials and methods

### Genes

Full-length genes encoding GtCCR1 (GenBank: LC591948), GtCCR2 (NCBI reference sequence: XM_005841372), GtCCR3 (NCBI reference sequence: XM_005833981), GtCCR4 (GenBank: MF039475), and GtCCR5 (NCBI reference sequence: XM_005827826) were chemically synthesized after human codon optimization (GenScript).

### Expression plasmids

Plasmid DNAs for expression in mammalian cells or primary cortical neuron culture, pGtCCR1-3.0-eYFP, pGtCCR2-3.0-eYFP, pGtCCR3-3.0-eYFP, pGtCCR4-3.0-eYFP, pGtCCR5-3.0-eYFP, phChR2-3.0-eYFP, pC1V1-3.0-eYFP, pChRGR-3.0-eYFP, pCaMKIIa-GtCCR4-3.0-eYFP and pCaMKIIa-hChR2-3.0-eYFP, were created by an in-fusion reaction by using the protein-coding sequences of GtCCR1-5. The successful insertion of each ChR gene into the vector plasmid was verified by DNA sequencing (Fasmac Co., Ltd., Kanagawa, Japan). Human codon optimized ChR2 (hChR2), C1V1 and ChRGR were kind gifts from Dr. H. Yawo (Tohoku University, Japan)^29,30^. PCR primers used for the reactions are summarized in Table S1.

### Virus preparation

AAV7m8 GtCCR4-Venus vector was produced by the helper-free triple transfection procedure and purified using affinity chromatography (GE Healthcare)^40^. Viral titers were determined by quantitative PCR using TaqMan technology (Life Technologies, Gaithersburg, MD, USA). The purity of the vectors was assessed by 4–12% sodium dodecyl sulfate-acrylamide gel electrophoresis and fluorescent staining (Oriole, Bio-Rad, Hercules, CA, USA). The transfer plasmid (pAAV-hSyn-GtCCR4-Venus-WPRE and pAAV-hSyn-ChR2-Venus-WPRE) was constructed by inserting the human synapsin promotor sequence and GtCCR4-Venus fragment with the WPRE sequence into an AAV backbone plasmid, respectively (pAAV-CMV, Stratagene, La Jolla, CA, USA).

### Cell culture

The electrophysiological assays of ChRs were performed on ND7/23 cells, hybrid cell lines derived from neonatal rat dorsal root ganglia neurons fused with mouse neuroblastoma^41^. ND7/23 cells were grown on a collagen-coated coverslip in Dulbecco's modified Eagle’s medium (Fujifilm Wako Pure Chemical Corporation, Osaka, Japan) supplemented with 2.5 μM all-*trans* retinal, 5% fetal bovine serum under a 5% CO_2_ atmosphere at 37 °C. The expression plasmids were transiently transfected by using FuGENE HD (Promega, Madison, WI, USA) according to the manufacturer’s instructions. Electrophysiological recordings were then conducted 16–36 h after the transfection. Successfully transfected cells were identified by eYFP fluorescence under a microscope prior to the measurements.

Cortical neurons were isolated from embryonic day 16 Wistar rats (Charles River Laboratories Japan, Inc., Kanagawa, Japan) using Nerve-Cells Dispersion Solutions (297-78101, Fujifilm Wako Pure Chemical Corporation) according to the manufacturer's instructions and grown in the neuron culture medium (148-09671, FUJIFILM Wako Pure Chemical Corporation) under a 5% CO_2_ atmosphere at 37 °C. The expression plasmids were transiently transfected in cortical neurons by calcium phosphate transfection at days in vitro (DIV) 5. Electrophysiological recordings were then conducted at DIV21–23 of neurons identified by fluorescence under a conventional epifluorescence system.

### Electrophysiology

All experiments were carried out at room temperature (22 ± 2 °C). Photocurrents and action potentials were recorded using an amplifier IPA (Sutter Instrument, Novato, CA, USA) under a whole-cell patch clamp configuration. Data were filtered at 5 kHz, sampled at 10 kHz, and stored in a computer (SutterPatch, Sutter Instrument, Novato, CA, USA). The standard internal pipette solution for whole-cell voltage-clamp recording contained (in mM) 110 N-methyl D-glucamine, 2 MgCl_2_, 1 CaCl_2_, 10 HEPES, 10 EGTA, 3 glucose, adjusted to pH 7.4 with HCl. The standard extracellular solution for whole-cell voltage-clamp recording contained (in mM) 140 NaCl, 2 MgCl_2_, 2 CaCl_2_, 10 HEPES, 11 glucose, adjusted to pH 7.4 with *N*-methyl d-glucamine. Time constants were determined by a single exponential fit unless noted otherwise.

The internal pipette solution for the whole-cell current-clamp recordings from cortical neurons contained (in mM) 125 K-gluconate, 10 NaCl, 0.2 EGTA, 10 HEPES, 1 MgCl_2_, 3 MgATP, 0.3 Na_2_GTP, 10 Na_2_-phosphocreatine, 0.1 leupeptin, adjusted to pH 7.4 with KOH. The extracellular Tyrode’s solution contained (in mM) 138 NaCl, 3 KCl, 10 HEPES, 4 NaOH, 2 CaCl_2_, 1 MgCl_2_, 11 glucose, adjusted to pH 7.4 with KOH. In all cortical neuron experiments, Tyrode’s solution contained 20 μM 6,7-dinitroquinoxaline-2,3-dione (DNQX, Tocris Bioscience, Ellisville, MO, USA), 25 μM d-(−)-2-amino-5-phosphonovaleric acid (D-AP5, Tocris), and 100 μM picrotoxin (Nacalai Tesque, Inc., Kyoto, Japan) to block all synaptic inputs. The directly measured liquid junction potential was 16.3 mV and was compensated.

### Animals, virus infection

Six week-old *rd1* mice (C3H/HeNSlc) were anesthetized with an intraperitoneal injection of ketamine and xylazine. Mice were then injected with 1 µL of the virus vector into vitreous (1.0 × 10^10^ vg/eye). Injections were made with a glass micropipette with a microinjection apparatus (IM 300 microinjection; Narishige, Tokyo, Japan). After 4 weeks, the electrophysiological measurement was performend.

### Electrophysiology: assay with the multi electrode array

All procedures were carried out in a dark room under 660 nm LED light. The mouse retina was removed from the eye lens, then incubated in AME’S medium (Sigma-Aldrich) at room temperature with continuous O_2_ supplementation until measurement. The retinal was positioned on a 64-multi channel electrode chamber. The electrical response was recorded by MEA2100 (Multi Channel Systems MCS GmbH, Reutlingen, Germany).

### Optics

For whole-cell patch clamp recording, irradiation at 470 or 530 nm was carried out using Colibri7 or collimated LED (Carl Zeiss, Oberkochen, Germany or parts No. LCS-0530-03-22, Mightex, Toronto, Canada) controlled by computer software (SutterPatch). Light power was directly measured at an objective lens of microscopy by a visible light-sensing thermopile (MIR-100Q, SSC Inc., Mie, Japan).

For the experiment with a multi-electrode array, an optical lens was mounted under a recording chamber. Illumination was carried out by using LED M530L4 (530 nm) (Thorlabs Japan Inc. Tokyo, Japan).

### Statistical analysis

All data in the text and figures are expressed as mean ± SEM and were evaluated with the Mann–Whitney *U* test for statistical significance, unless otherwise noted. It was judged as statistically insignificant when *P* > 0.05.

### Ethical approval

This study was carried out in accordance with regulations by the institutional animal care and use committee of the Nagoya Institute of Technology (approval number: 2020001), and by the Nagoya University animal experiment committee (approval number 20109) and this study was also approved by both these committee. All methods are reported in accordance with the ARRIVE guidelines.

## Supplementary Information

Supplementary Information.

## Data Availability

All materials and data will be made available to readers upon reasonable request to Satoshi Tsunoda (tsunoda.satoshi@nitech.ac.jp) or (Hideki Kandori kandori@nitech.ac.jp), without undue qualifications.
