# Supplementary material for: A light-gated cation channel with high reactivity to weak light
Source: Sci Rep. 2023 May 10;13:7625. doi: 10.1038/s41598-023-34687-7 (PMC10172181; doi:10.1038/s41598-023-34687-7)
Supplement: Supplementary file 1 — Supplementary Information. [file 41598_2023_34687_MOESM1_ESM.pdf]

Supplementary Information

# A light-gated cation channels with high-reactivity to weak light


Authors

Shoko Hososhima[1,2], Shinji Ueno[3,4], Satoshi Okado[3], Ken-ichi Inoue[5], Masae Konno[1,2], Yumeka Yamauchi[1], Keiichi Inoue[1,2], Hiroko Terasaki[3], Hideki Kandori[1,2]†, Satoshi P. Tsunoda[1,2†]

Affiliations

1. Department of Life Science and Applied Chemistry, Nagoya Institute of Technology, Showa-ku, Nagoya, Aichi 466-8555, Japan.
2. OptoBioTechnology Research Center, Nagoya Institute of Technology, Showa-Ku, Nagoya, Aichi 466-8555, Japan
3. Department of Ophthalmology, Nagoya University Graduate School of Medicine, 65 Tsurumai-cho, Showa-ku, Nagoya, Aichi, 466-8550 Japan.
4. Department of Ophthalmology, Hirosaki University Graduate School of Medicine, 5,Zaifu-cho, Hirosaki, Aomori, 036-8562, Japan
5. Primate Research Institute, Kyoto University, Inuyama, Aichi 484-8506, Japan

Fig.S1

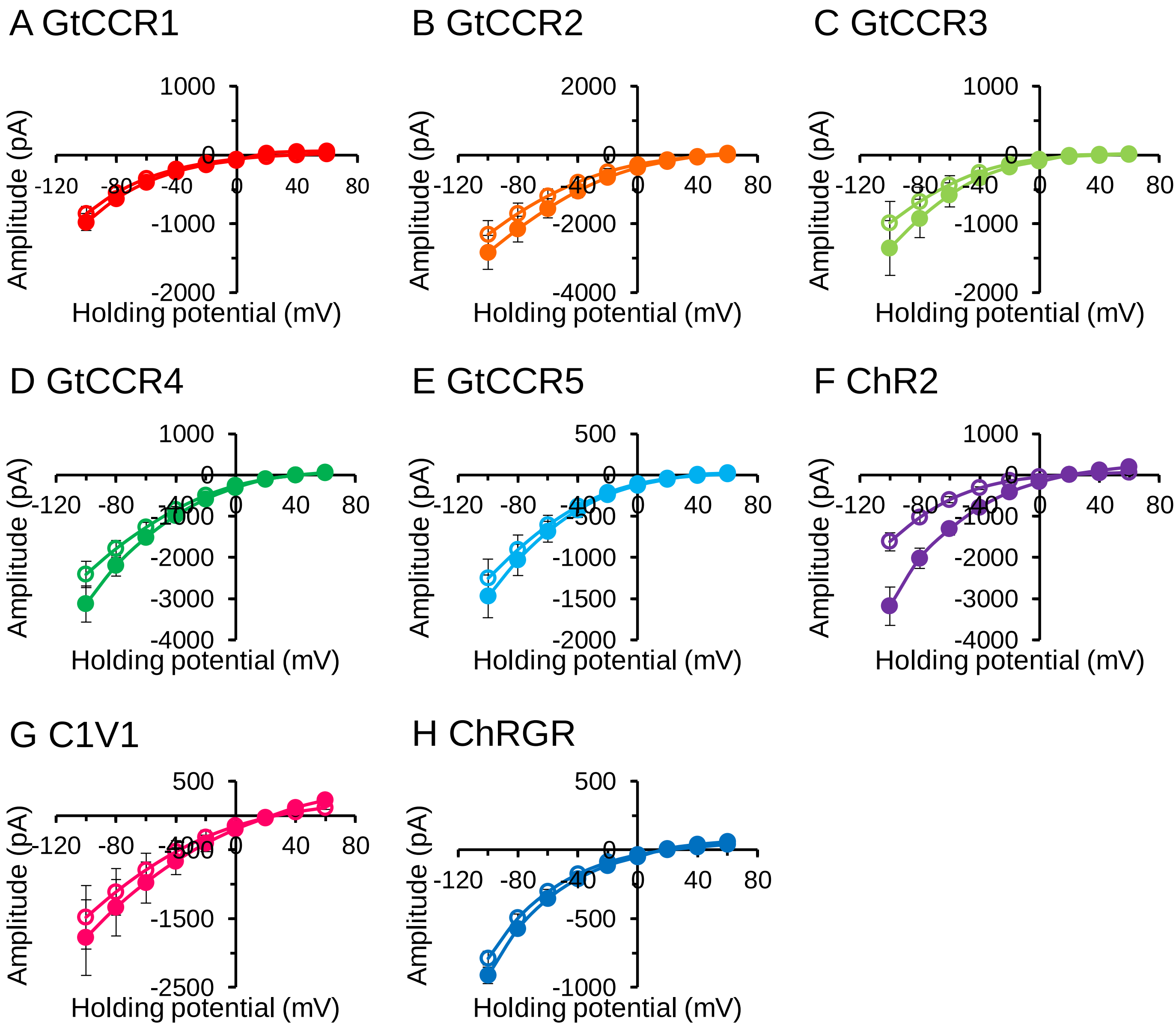


**Figure S1: Current-voltage relation (I-V plot) of all channelrhodopsins tested in this study.** Filled circle: the transient peak current component. Open circle: steady state current component. The internal pipette solution for whole-cell voltage-clamp contained (in mM) 110 N-Methyl D-glucamine, 2 $MgCl_2$, 1 $CaCl_2$, 10 HEPES, 10 EGTA, 3 glucose, adjusted to pH 7.4 with HCl. The extracellular solution for whole-cell voltage-clamp contained (in mM) 140 NaCl, 2 $MgCl_2$, 2 $CaCl_2$, 10 HEPES, 11 glucose, adjusted to pH 7.4 with N-Methyl D-glucamine.

Fig.S2

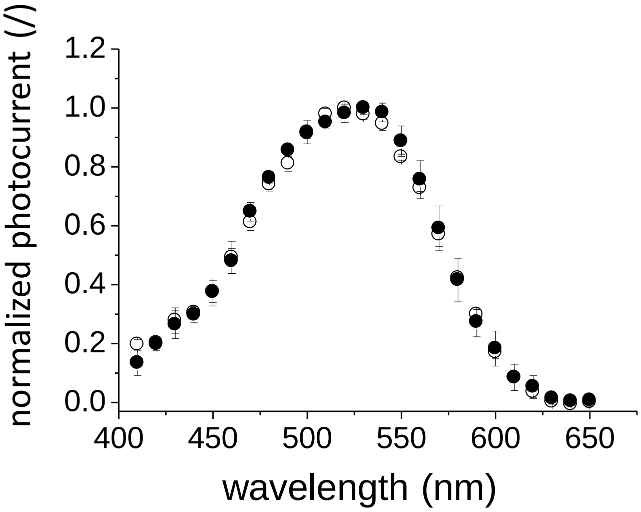


**Figure S2: Action spectra of GtCCR4 and GtCCR5**

Wavelength dependency of the photocurrent from GtCCR4 (open circle) and GtCCR5 (filled circle) was depicted. Membrane voltage was clamped at -40 mV. Standard solutions were used.

Fig.S3

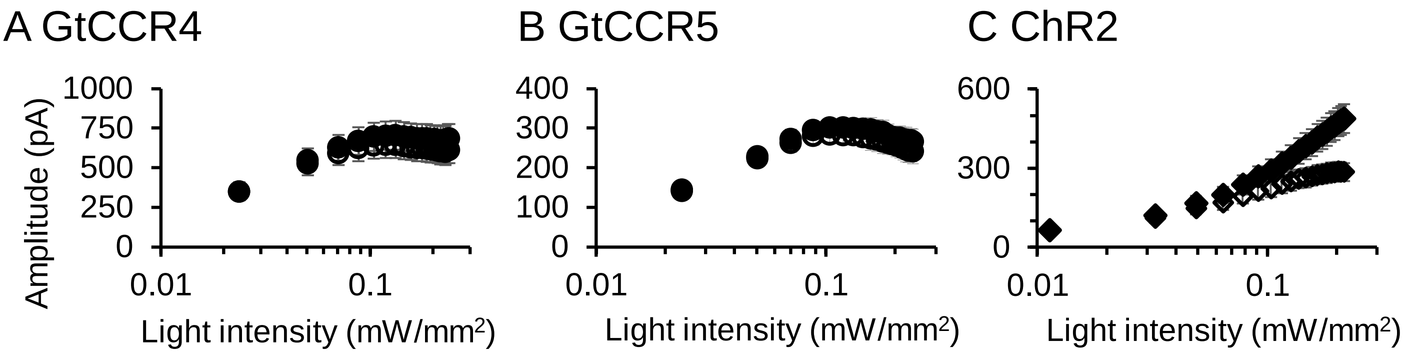


**Figure S3: Light intensity dependency of photocurrent from GtCCR4 (A), GtCCR5(B) and ChR2(C)**

Photocurrent at -60 mV was measured under weak light power. 530 nm light (A, B) and 480 nm light (C) was used. Filled symbol: transient peak component. Open symbol: steady state component. Standard solutions were used.

Fig.S4

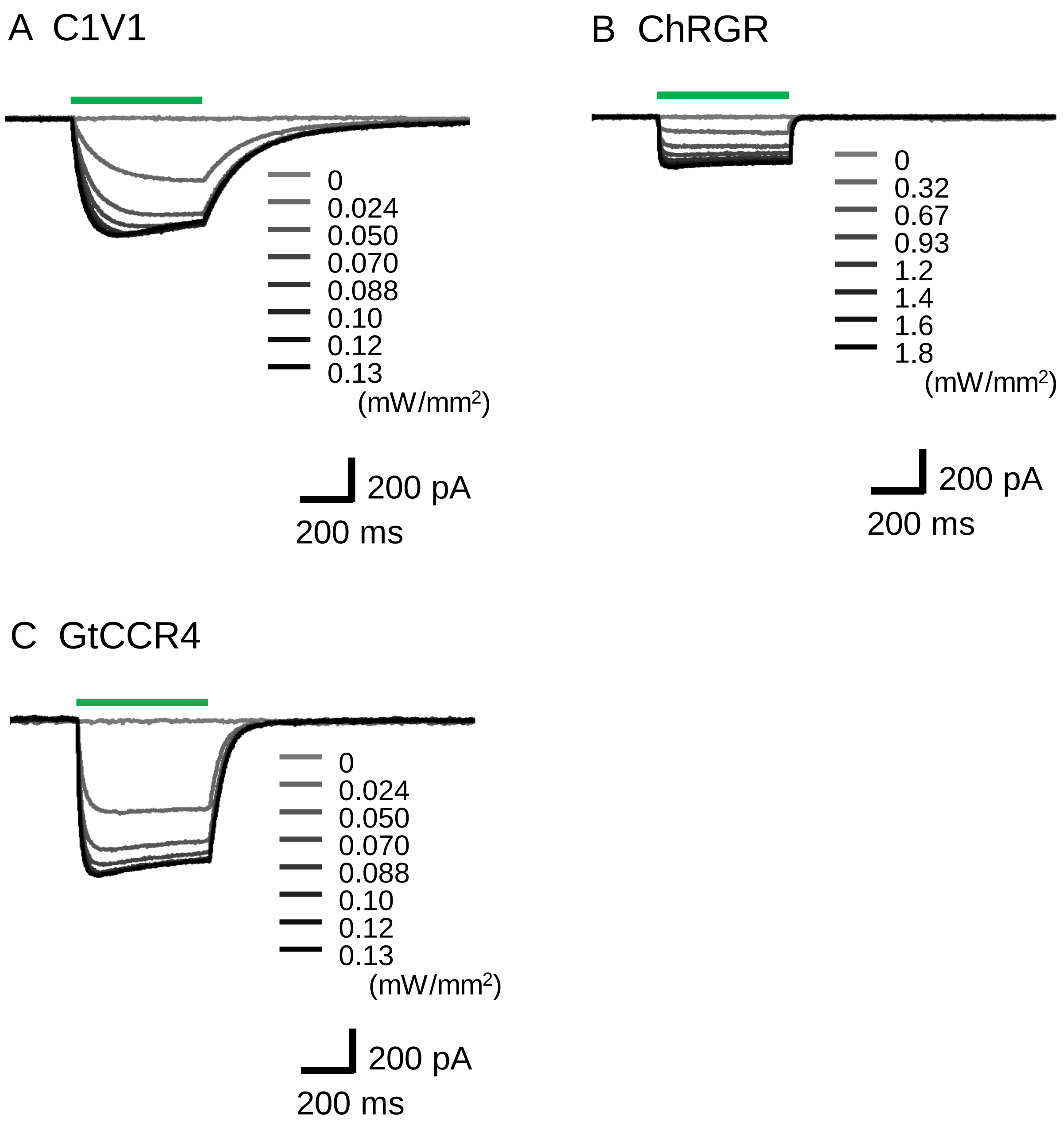


**Figure S4: Photocurrent traces of C1V1 (A), ChRGR(B) and GtCCR4(C) by weak light range.** Voltage clamp recordings at -60 mV are shown. Various intensity of 530 nm light as indicated on each panel was illuminated during the time as shown by a green. Standard solutions were used.

Fig.S5

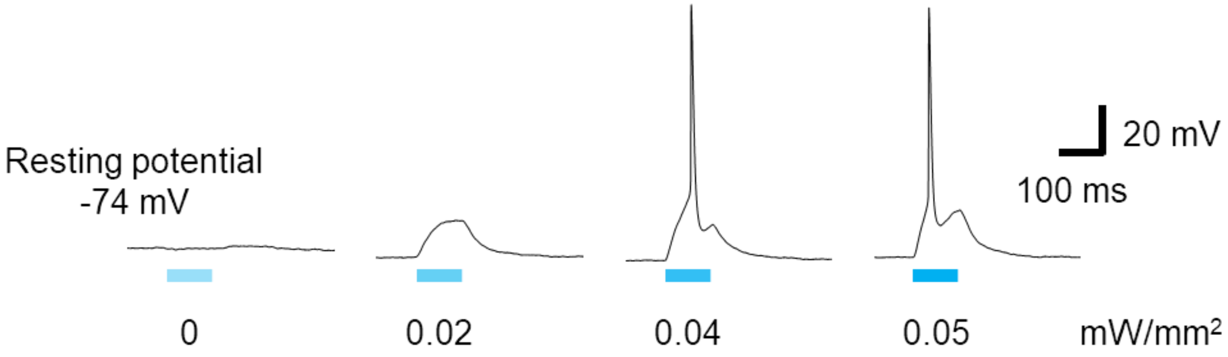


**Figure S5: Optical stimulation of ChR2-expressing neuron.**

Representative current clamp traces of a ChR2-expressing preculture neuron are shown. 470 nm light in various intensities indicated under each trace was illuminated for 100 ms.

Fig.S6

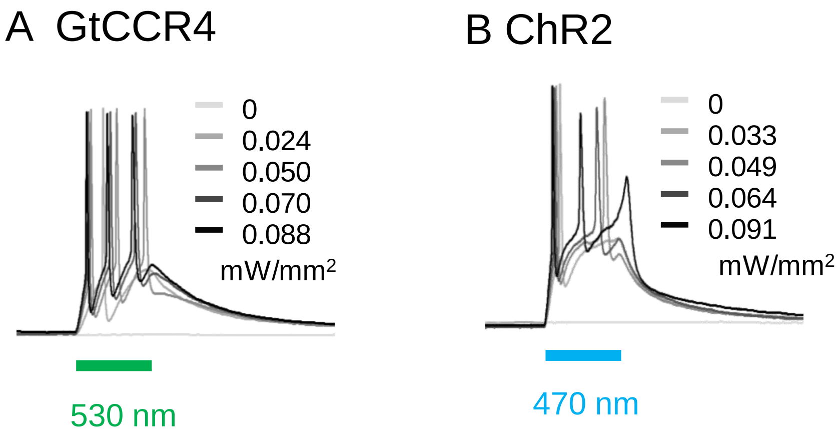


**Figure S6: Light power dependency of GtCCR4 (A) and ChR2 (B)-expressing neurons.** Representative current clamp traces of a preculture neuron are shown. Light in various intensities indicated under each trace was illuminated for 100 ms.

Fig.S7

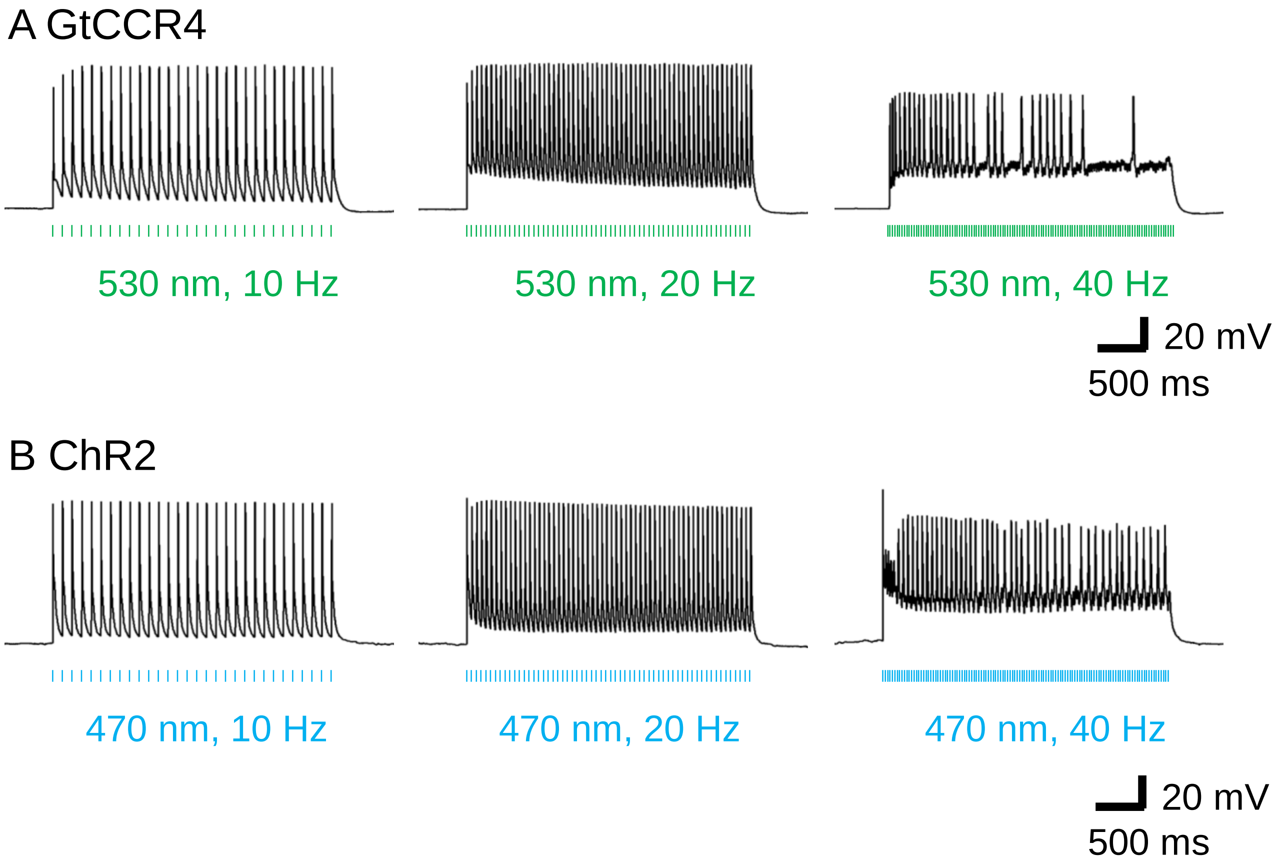


**Figure S7: Frequency response of GtCCR4 (A) and ChR2 (B)-expressing neurons.** Representative current clamp traces of a GtCCR4- or a ChR2-expressing preculture neuron are shown. Neurons were stimulated by 530 nm (A) or 470 nm (B) light at 5, 20 or 40 Hz as indicated under each panel.

Table S1  Primer list used for PCR

| | | Sense primers | Anti-sense primers | Template |
|---|---|---|---|---|
| **pGtCCR4-3.0-eYFP** | Insert | gctctttggatccccaacggcctcggactc | gctctttggatccccaacggcctcggactc | pGtCCR4-EGFP |
| | Vector | EcoRI, BamHI | | pDdNaR-3.0-eYFP |
| **phChR2-3.0-eYFP** | Insert | EcoRI, BamHI | | phChR2-Venus |
| | Vector | EcoRI, BamHI | | phKR2-3.0-eYFP |
| **pGtCCR4-P2A-Venus** | Insert | Synthesized gene (Azenta Life Sciences) | | |
| | Vector | gaagctgatctgcaccaccggcaagctgc | tcctgatccggtggcgaccggtggatc | pGtCCR4-Venus |
| **pCaMKIIa-GtCCR4-3.0-eYFP** | Insert | EcoRI, BamHI | | pGtCCR4-3.0-eYFP |
| | Vector | EcoRI, BamHI | | pCaMKIIa-hKR2-3.0-eYFP-WPRE |
| **pCaMKIIa-hChR2-3.0-eYFP** | Insert | EcoRI, BamHI | | phChR2-Venus |
| | Vector | EcoRI, BamHI | | pCaMKIIa-hKR2-3.0-eYFP-WPRE |
| **pCaMKIIa-GtCCR4-P2A-Venus** | Insert | cttcgaattcgccaccatgacgacgtctgcccctc | gattgcggccgctcattacttgtacagctcgtcc | pGtCCR4-P2A-Venus |
| | Vector | tgagcggccgcaatcaacctctg | ggtggcgaattcgaagcttgagc | pCaMKIIa-GtCCR4-3.0-eYFP |
